# Women’s experience with a first- and second-degree perineal tear and episiotomy – a qualitative study

**DOI:** 10.1186/s12884-025-08369-3

**Published:** 2025-11-07

**Authors:** Katrine Aasekjær, Sunneva Skjerven, Åse Marie Bratlien, Cecilie Volnes, Linn Marie Sørbye

**Affiliations:** 1 Western Norway University of Applied Science, Bergen, Norway; 2 Haukeland University Hospital, Bergen, Norway

**Keywords:** Perineal tear, Post-partum period, Systematic follow up, Affirmation and psychological support, Qualitative research.

## Abstract

**Background:**

Perineal tears are associated with both physical- and psychological complications, and studies have found that women’s individual experience of the pain from the tear does not necessarily reflect the extent and severity of the tear. Women seem to experience a lack of consistency and sufficient follow up in the post-partum period related to perineal trauma. The aim of this study was to explore women’s experiences with first- and second- degree perineal tears and episiotomy in the post-partum period in an outpatient perineal clinic at a tertiary hospital in Norway.

**Materials and methods:**

We conducted a qualitative study using individual semi-structured interviews, with women experiencing a follow-up of their perineal tear from an outpatient clinic. Participants were recruited from one tertiary hospital in Norway, and we conducted 18 individual interviews. All interviews were audio recorded, transcribed verbatim and analysed using systematic text condensation as described by Malterud.

**Results:**

Three code groups and one overarching theme were identified. The overarching theme was that the women experienced a feeling of being left alone with the responsibility of both their physical and psychological needs regarding their perineal tear. The women described (I) lack of affirmation and emotional support, (II) lack of guidance on normal processes and (III) lack of systematic follow up of perineal tears.

**Conclusion:**

The women in this study felt they were left alone with their perineal tear and did not receive support nor recognition from healthcare professionals regarding the challenges they faced. The physical and psychological consequences women experienced related to their perineal tear highlight the need for a more systematically and individually tailored care for women in the post-partum period, from both midwives and doctors.

## Background

Perineal tears related to vaginal delivery appear in almost 85% of all first-time mothers [1] and are associated with physical - and psychological complications. A frequent reported concern following a perineal tear is perineal pain [1] which influences the woman’s post-partum period by reducing her ability to perform daily activities, and to find comfortable positions during breastfeeding [2–6]. Also, women describe a delay in attachment to their baby and a feeling of not meeting their expectations to motherhood [4, 6, 7].

The most prominent reported complications are perineal pain, dyspareunia, and stress incontinence [2]. For some women, complications persist and negatively continue influence their relationship with both their baby and partner [2–4, 8–10]. However, women’s individual experience of the pain and the perceived severity of the tear do not necessarily reflect the grading of the tear [3–5, 8, 9].

Studies report that women are unprepared for the physical and psychological discomfort and pain the perineal tear causes them in the post-partum period. Also, experiencing complications from a perineal tear could make women anxious for future pregnancies and childbirth [4, 11–13]. Knowledge on how perineal tears impact women’s health and well-being in the post-partum period is scarce. Also, most studies have investigated complications related to third – and fourth degree tears, with less focus on first- and second-degree tears. Despite evidence-based guidelines and recommendations on perineal care, women seem to experience a lack of consistency and sufficient follow up in the post-partum period [14]. The main objective of this study was to explore women’s experiences with first – and second-degree perineal tears and episiotomy in the post-partum period in an outpatient perineal clinic at a tertiary hospital in Norway.

## Methods

### Study design

In this qualitative study with individual semi-structured interviews, we analysed data trough systematic text condensation as described by Malterud [15]. We conducted a purposive sample [16], and participants received oral- and written information about the study prior to participation. Ethical approval to conduct the study was obtained from the Regional Medical and Health Research Ethics Committee in Norway (reference number 284944) and the Data protection officer at the University Hospital evaluated the study.

### Study setting

The study was carried out at in an outpatient clinic that offers post-partum follow-up of perineal tears at a tertiary hospital in Norway with. The majority of women visiting the clinic have a first or second-degree perineal tear with or without an episiotomy. The actual hospital does not offer a systematic post-partum follow-up program to women with perineal tears if the sphincter muscle is not affected. However, women with a third - or fourth- degree perineal tear, are part of a systematic post-partum follow-up program and are therefore not by default referred to the outpatient clinic. Most women visited the outpatient clinic 1–2 weeks following childbirth, but some women did not seek help for their perineal problems until up to nine months after giving birth. The number of follow-up consultations at the outpatient clinic vary and is related to the perineal complication’s women experience. The first consultation is free of charge, after which the women must cover a user fee of 32 euro for each subsequent follow-up appointment. Women can be referred to the outpatient clinic from the postpartum ward at the hospital, by a midwife in the municipal health service, by their general practitioner, or by taking personal initiative.

### Participants

Women eligible for the study were recruited at the time of their appointment at the outpatient clinic. A total of 92 women had a follow-up appointment during the study period, all of whom were invited to participate in this qualitative study. Finally, 18 women consented to participate in individual interviews. The main reason for not participating was considerable challenges with the perineal tear which made women feel unable to participate. After 18 interviews we also considered that further interviews no longer contributed to new insights and that sufficient informational power was met [17].

### Data collection

Data were collected from September 1 st to December 31 st, 2022. We used a semi-structured interview guide with open-ended questions informed by the existing research literature, in addition to the authors clinical and methodological experience. The interview guide had a chronological structure, aiming to explore the women’s experiences with their perineal tear in the time period following childbirth and until they were offered help from the outpatient clinic. We used the same interview guide for all participants, however, the depth of exploration into various topics differed among the participants. Each interview took place in a private environment, chosen by the participant. Hence, most interviews were conducted digitally using the Zoom via Uninett. Individual interviews were audio-recorded, transcribed verbatim and stored at the local hospital’s research server. Each transcript was checked by the researchers for accuracy against the original recording before the data analysis.

### Data analysis

**Fig. 1:**
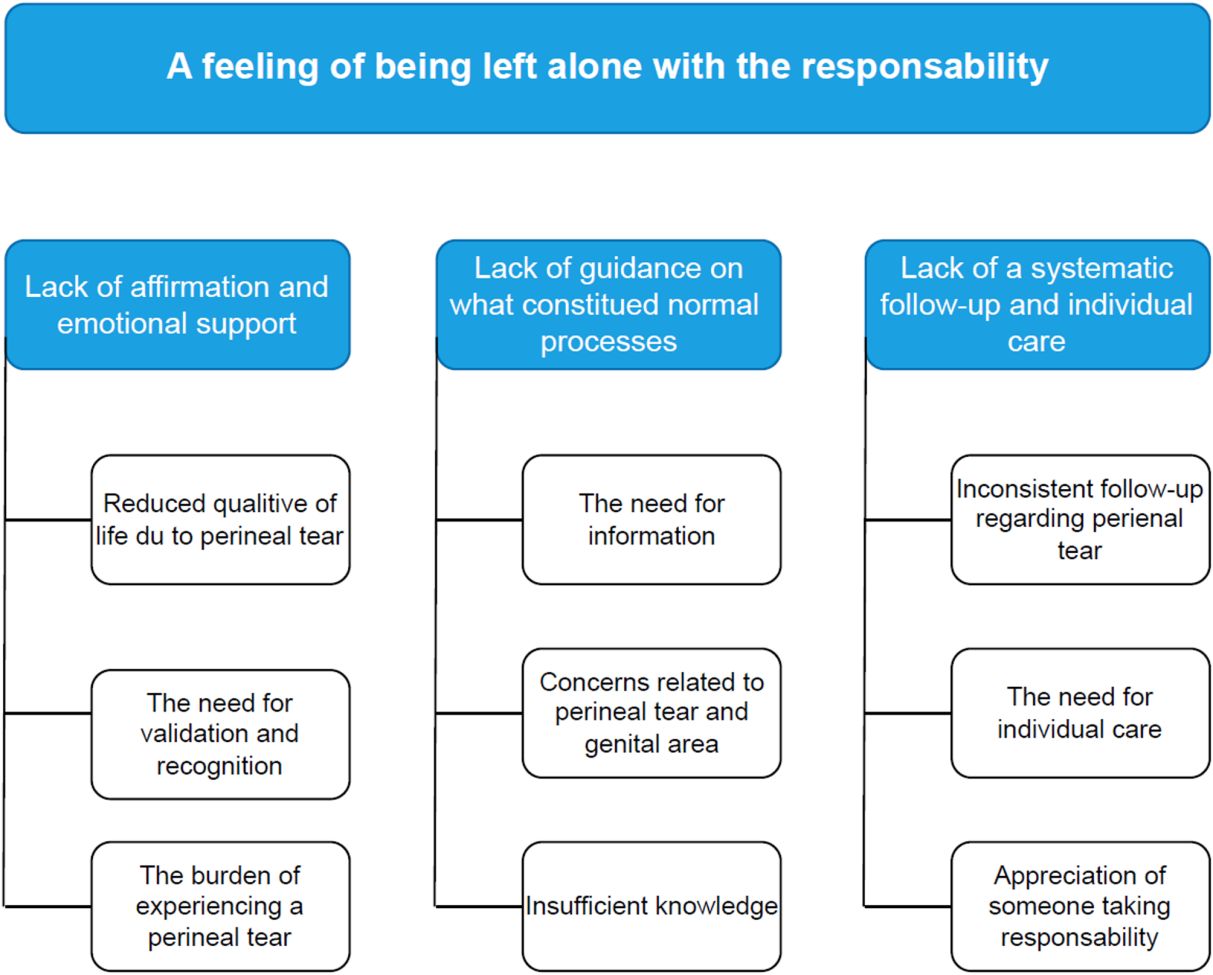
Women’s experience with a first- and second-degree perineal tear and/or episiotomy, illustrated with the overarching theme, and code groups with associated subgroups

Data were analysed using systematic text condensation as described by Malterud [15]. This inductive and pragmatic analytical method for thematic cross-sectional analysis of qualitative data was carried out following the four steps of the systematic text condensation [15]. In the first step the transcripts were read and re-read to get an overall impression of the data identifying themes. In the second step, we identified meaning units describing the women’s experiences related to their perineal tear in the post-partum period. Once the meaning units had been identified the third step was to sort the meaning units into different code groups. In this step, we abstracted the content of the code groups of meaning units and formed subgroups describing different aspects of the content of the code groups. This was a flexible process where we went back and forth in the original data material aiming to identify and organize data elements in line with the aim of the study (Fig. 1). In the fourth and final step, we used text condensates from step three as the basis for our final analytic text. Direct quotations were used to illustrate each code group.

After assessing the data independently, the first and second author reached consensus before including the third and fourth author in the discussion. All authors took part in all steps of the analysis. Data were reported using consolidated criteria for reporting qualitative studies (COREC) 32-items checklist [18].

## Results

A total of 18 women participated in the study. The most common reasons for contacting the outpatient perineal clinic were perineal pain, ruptured sutures, excessive growth of granulation tissue or infection.

The overarching theme identified in the analysis was that women experienced a feeling of being left alone with a responsibility of both their physical and psychological needs regarding their perineal tear. Three code groups revealed that the women experienced (I) lack of affirmation and emotional support, (II) lack of guidance on what constituted normal processes, and (III) lack of a systematic follow-up and individual care.

### A feeling of being left alone with the responsibility

Women described a feeling of being left alone with the responsibility of both their physical and psychological challenges following their perineal tear. Pain from the perineal tear negatively influenced women’s interaction with their child and partner, in addition to restricting their daily activities. The women expressed concern about their future health, sexual functioning and had negative thoughts regarding future pregnancies and childbirth opportunities. Uncertainty on what to expect, combined with an inconsistent health-care follow-up, reinforced their sense of being unsupported and left alone. The women requested a need for more information, to be acknowledged for their problems, and for a more individual and tailored care of their perineal tear. A combination of women’s experiences and needs, along with the perception of inadequate follow-up, gave them a feeling of being responsible alone in managing their recovery. As one woman described:

*“I received no explanation [about the perineal tear]. It felt as if nobody took the time to explain the situation. The only information I received was that I had a second-degree tear and that I should consider myself lucky. However*,* there was no guidance provided on how I should properly care for my tear” (Interview 4)*.

#### Lack of affirmation and emotional support

Women described the physical discomfort from the perineal tear as a psychological burden, due to the lack of affirmation and support from health-care professionals in the post-partum period. Women described the period after giving birth as mentally exhausting, as their concerns were often dismissed or not acknowledged, leaving them with a feeling that their problem was not of importance. Irrespective of the severity of the tear or the duration of the pain, it had an impact on the women’s daily lives. The women found themselves spending a considerable time lying down, as sitting comfortably was impossible. This restricted breastfeeding to lying or standing positions. Also, pain and the fear of losing control over their bowel function in public seemed to isolate women in their homes. Physical challenges were described by the women as affecting their attachment to the child, because the pain influenced their ability to engage and being close to their child in a way they desired. Also, women experienced a dependency on extra support and help from their partner, who had to take responsibility for both mother and child, and in turn negatively which influenced their relationship.

*“The postpartum period was incredibly difficult due to the challenges caused by the tear. I felt like I missed out on the opportunity to bond [with my baby]. It was not at all the experience I had imagined” (Interview 3)*.

Women spent considerable time reflecting on why early postpartum period had unfolded as it did and questioned if they could have done things differently. Women expressed concerns regarding their future sexual life and reproductive health and had a fear about their bodies being permanently damaged. The worries concerning future sexual life, did not take away the wish of a future pregnancy, but the women feared complications such as uterine prolapse, ruptured sutures and infections. As one woman put it:

*“I remember thinking to myself that I might never have the desire to engage in sexual activities again because of the overwhelming pain. How would it ever heal properly? There was this constant fear that I would be permanently damaged”* (Interview 16).

#### Lack of guidance on what constituted normal processes

Although the women knew that experiencing a perineal tear was common, they had insufficient knowledge on normal wound healing and felt unprepared for the extent of pain they experienced. During pregnancy, neither doctors nor midwives discussed perineal tear and possible complications as a preparation for labour and the post-partum period. This lack of information continued after delivery and during their stay at the maternity ward and often led to uncertainty in defining what was normal processes and what deviated from normality. Since healthcare professionals seldom initiated conversations about perineal tears, women had an impression that the topic was taboo or inappropriate to discuss. Women expressed a desire for more information during pregnancy and upon discharge from the maternity ward, preferring a combination of oral and written guidance. They had a wish to understand the healing process and to have their tear assessed by professionals, as self-monitoring was difficult and often led to more worries. The absence of clear information contributed to delays in seeking help. As several women described:

*“The information you get in the postpartum ward is the questions you ask yourself. You get answers*,* but you have to ask the right questions. If you don’t recognise what you don’t know*,* it’s difficult. I didn’t realize I needed knowledge about the perineal tear. If I had received better information*,* I might have realized that it wasn’t normal. I endured six weeks of pain*,* but I could have received help after two weeks " (Interview 2 and 4)*.

#### Lack of systematic follow-up and individual care

Women described the follow-up care for perineal tears as inconsistent and lacking structure. Some received oral guidance, others were handed a leaflet containing general information without explanation. The opportunity for examination before discharge varied; some were offered one, others had to request for it, and some did not receive an examination at all. Also, women in the study felt that the six-week postpartum checkup with their general practitioner (GP) failed to meet their expectations and needs. There was no room to discuss their perineal tear, and the women described that their GP lacked the expertise to assess the perineal tear properly. Women expressed a clear preference for follow-up by a midwife or gynaecologist with specialized knowledge who could acknowledge their concerns and offer tailored support. The lack of a systematic follow-up from healthcare professionals, was finally provided when they attended the outpatient clinic where the women felt validated by professionals with expertise in perineal injuries. The women expressed gratitude for this care, although they wished it had come sooner. As one woman explained:

*“The follow-up at outpatient clinic has been fantastic. They explained everything*,* showed me the tear with a mirror*,* and I was re-sutured. The way they met me with support and information telling me that everything would heal and that they had experienced this many times before. Yes*,* it was painful*,* but knowing that it would be fine again was so important to me” (Interview 12)*.

## Discussion

Women described a feeling of being left alone with the responsibility of both their physical and psychological challenges of their perineal tear. This responsibility manifested itself by the women’s feeling of a fragmented follow-up and a health care system not able to give women affirmation nor emotional support on the problems and pain they experienced. The feeling of being left alone also revealed itself in the lack of guidance and continuity of essential postnatal care. The women experienced a discrepancy on their need for knowledge on how to care for their perineal tear, and the actual care and information they received from health care professionals. Women reported satisfaction and gratitude with the follow-up from the outpatient clinic where they finally were met by midwifes with specialized expertise who acknowledged and validated their challenges, given in a systematic and compassionate manner.

Women in our study expressed a need for better systematic follow-up given with affirmation and support. Women need to be met with compassionate care from healthcare professionals for managing the transition to motherhood [19]. Although the women were aware that perineal tears often occur during childbirth, they were unprepared for the extent of pain and complications that followed. Our findings revealed an unwarranted variation in the post-natal care and follow-up, which gave the women a feeling of being left alone with the responsibility for their own healthcare. The women described a health care system lacking a systematic focus on perineal care post-partum and providing limited information about healing expectations. Absence of information created insecurity amongst the women and reinforced the perception that they had to take responsibility for their own health and well-being. According to maternity care guidelines, women and their families should expect individual and evidence-based post-natal care and information [20, 21]. Research also shows that the transition to parenthood is improved when women are supported with consistent and trustworthy information from healthcare professionals [22]. Due to the perceived lack of information and follow-up, women worried about long-term effects on sexual health and future pregnancies. These concerns became an emotional strain, affecting their attachment and relationship to both their newborn and partner both of which are acknowledged as key psychological aspects in the transition to parenthood and the construction of a new family [19, 22, 23].

Women with perineal tear expressed an explicit need for more information on normal wound healing and future sexual health and reproduction. Our findings suggest that more and systematically attention on perineal care should be offered women in the post-natal period and integrated in the post-natal care. According to WHO continuity of care is an important goal in achieving positive childbirth experience for all women and involves the postnatal period [20]. Continuity refers to care provided by the same professional over time, yet women in our study reported a lack of consistency in both information and emotional support. Women expressed that if they had received systematic information and follow-up given in a respectful manner from healthcare providers, their experience might have been easier and better prepared them for the postnatal period. Studies have shown that women and their partners hold specific expectations for the postnatal period. However, expectations regarding the maternal role, the newborn, and the partner are often disrupted by the challenges associated with a perineal tear [2, 5, 8, 12, 24–26]. Findings from previous studies are in line with our results where women described the postnatal period as emotionally demanding and marked by feelings of inadequacy in their roles as both parent and partner.

Maternity care in Norway holds high quality, hence surveys of the post-natal care characterize this care as fragmented, less individualized, and lacking continuity [13]. Affirmative and emotional support are important for women in the postnatal period because it reduce stress and increase coping skills in the transition to motherhood [19, 20]. Consistent information given by competent healthcare providers, fosters both emotional reassurance and practical guidance. It is of importance that information and follow up is offered by specialised healthcare providers. Women benefit from relevant, individualized, and predictable support [22, 23]. The women in our study confirm these aspects, reporting a lack of support and acknowledgment from healthcare providers which reinforced women with a feeling of being left alone with a responsibility they were not equipped to manage.

### Clinical implications

Findings from this study highlight the importance of identifying, acknowledge and responding to women’s needs following a perineal tear, regardless of the extent of the perineal tear. Healthcare professionals, particularly midwives, play a crucial role in offering systematic and individualized follow-up care that affirms and supports women both physically and emotionally.

To improve postnatal care, women need clear written and verbal information at discharge, including guidance on expected healing, pain management, and when and where to seek help. Midwives should be trained to actively address perineal concerns during routine postnatal visits, creating an environment for open dialogue and emotional support. Perineal health should be included in antenatal education to prepare women for potential outcomes and prepare them with coping strategies. By implementing these measures, healthcare systems can better support women in their transition to motherhood.

Women need affirmation, knowledge and reassurance from healthcare professionals on how to manage their perineal tear, to have a positive postnatal experience. A positive postnatal experience is of relevance for future pregnancies and women’s health [19].

### Strengths and limitations

Women were recruited from an outpatient clinic at tertiary hospital in Norway. A purposive sample ensured an in-depth understanding of the phenomena of interest, investigating the experience from a total 18 women. It is important to note that complications and experiences related to perineal trauma often are underreported by women, hence the topic is associated with privacy and sensitivity. Therefore, this study adds important knowledge to the field.

Informational power was reached providing a comprehensive understanding of the women’s experience with first- and second-degree perineal tears. The recruitment relied on self-selection, meaning that our findings are based on the experience from participants who are willing to share their insights. However, our findings align with those from larger studies [1, 9].

Our sample represents a selected group of women referred to an outpatient clinic. The participants may differ from the broader population of postpartum women in terms of symptom severity, help-seeking behaviour, or access to care. Second, sociodemographic data such as educational background, ethnicity, and language proficiency were not systematically collected, which restricts our ability to assess how these factors may have shaped the women’s experiences. The study was conducted at a single tertiary hospital in Norway, and the organizational context may differ from other clinical settings. Still, the finding from this study may be relevant to a broader population of postpartum women, and the women’s voices in this study may identify unmet needs for women with perineal tears. Finally, the aim of this study was not to generalize but to give an in-depth knowledge and understanding of women’s experience in line with the aim of a qualitative study.

All authors are midwifes either working as midwifes or researchers. The professional experience from the authors provided valuable insight during the data collection and analysis. To maintain reflexivity the authors followed a systematic and transparent process, were codes, themes and meaningful units were discussed continuously, resulting in meaningful interpretation of data.

## Conclusion

Women described a feeling of being left alone with the responsibility of both their physical and psychological needs regarding their perineal tear. Three sub-themes revealed that women experienced a lack of affirmation and emotional support regarding their perineal tear, lack of guidance on what constituted normal processes, and a lack of a systematic follow-up and individual care of their perineal tear. Women expressed that they did not receive support nor recognition from healthcare professionals regarding the challenges they faced. The physical and psychological consequences women experienced from their perineal tear demonstrates that there is a need for systematic and individualized post-natal care. Healthcare providers should offer clear written and verbal guidance at discharge, covering expected healing, pain management, and when to seek help. These measures can reduce emotional strain, strengthen coping, and promote a more positive transition to motherhood.

## Data Availability

The data that support the findings of this study are available on request from the corresponding author. The data are not publicly available due to privacy or ethical restrictions.
